# GABA Measurement in a Neonatal Fragile X Syndrome Mouse Model Using ^1^H-Magnetic Resonance Spectroscopy and Mass Spectrometry

**DOI:** 10.3389/fnmol.2020.612685

**Published:** 2020-12-18

**Authors:** Samantha T. Reyes, Sanaz Mohajeri, Karolina Krasinska, Scarlett G. Guo, Meng Gu, Laura Pisani, Jarrett Rosenberg, Daniel M. Spielman, Frederick T. Chin

**Affiliations:** ^1^Department of Radiology, Stanford University, Stanford, CA, United States; ^2^Stanford University Mass Spectrometry Laboratory, Stanford University, Stanford, CA, United States

**Keywords:** fragile X syndrome, Fmr1 knockout, mouse, neonatal, GABA, ^1^H-MRS, LC-MS/MS

## Abstract

Fragile X syndrome (FXS) is the leading monogenetic cause of autism spectrum disorder and inherited cause of intellectual disability that affects approximately one in 7,000 males and one in 11,000 females. In FXS, the *Fmr1* gene is silenced and prevents the expression of the fragile X mental retardation protein (FMRP) that directly targets mRNA transcripts of multiple GABA_A_ subunits. Therefore, FMRP loss adversely impacts the neuronal firing of the GABAergic system which creates an imbalance in the excitatory/inhibitory ratio within the brain. Current FXS treatment strategies focus on curing symptoms, such as anxiety or decreased social function. While treating symptoms can be helpful, incorporating non-invasive imaging to evaluate how treatments change the brain’s biology may explain what molecular aberrations are associated with disease pathology. Thus, the GABAergic system is suitable to explore developing novel therapeutic strategies for FXS. To understand how the GABAergic system may be affected by this loss-of-function mutation, GABA concentrations were examined within the frontal cortex and thalamus of 5-day-old wild type and *Fmr1* knockout mice using both ^1^H magnetic resonance imaging (^1^H-MRS) and liquid chromatography-tandem mass spectrometry (LC-MS/MS). Our objective was to develop a reliable scanning method for neonatal mice *in vivo* and evaluate whether ^1^H-MRS is suitable to capture regional GABA concentration differences at the front end of the critical cortical period where abnormal neurodevelopment occurs due to FMRP loss is first detected. ^1^H-MRS quantified GABA concentrations in both frontal cortex and thalamus of wild type and *Fmr1* knockout mice. To substantiate the results of our ^1^H-MRS studies, *in vitro* LC-MS/MS was also performed on brain homogenates from age-matched mice. We found significant changes in GABA concentration between the frontal cortex and thalamus within each mouse from both wild type and *Fmr1* knockout mice using ^1^H-MRS and LC-MS/MS. Significant GABA levels were also detected in these same regions between wild type and *Fmr1* knockout mice by LC-MS/MS, validating that FMRP loss directly affects the GABAergic system. Thus, these new findings support the need to develop an effective non-invasive imaging method to monitor novel GABAergic strategies aimed at treating patients with FXS.

## Introduction

Fragile X syndrome (FXS) affects approximately one in 7,000 males and one in 11,000 females (Hunter et al., 2014) and is the leading monogenetic cause of autism spectrum disorder (ASD) and inherited cause of intellectual disability (Lozano et al., 2014; Hagerman et al., 2017). Some features of FXS include attention-deficit/hyperactivity disorder (ADHD), social anxiety, and seizures (Hagerman et al., 2017). FXS is caused when there is an expansion of over 200 CGG-trinucleotide repeats in the 5′ region on the *FMR1* gene and hypermethylation of the gene occurs. This hypermethylation silences the *FMR1* gene, inhibiting the production of fragile X mental retardation protein (FMRP; Tabolacci et al., 2016). FMRP is an RNA-binding protein that is associated with translation of various mRNA relating to neuronal development (Darnell et al., 2011; Ascano et al., 2012; Maurin et al., 2018), regulation of ribosome stalling, neural circuit function, and synaptic plasticity (Gatto and Broadie, 2009; Zukin et al., 2009; Darnell et al., 2011). The loss of FMRP has also been associated with a downregulation of gamma-aminobutyric acid (GABA) membrane transporters and receptor scaffolding proteins (Gao et al., 2018). Furthermore, dysregulation of synaptic proteins is thought to disrupt synapse maturation (Remmers and Contractor, 2018) leading to long, thin dendritic spines seen in FXS (Irwin et al., 2000).

FMRP loss has been shown to affect many systems including the glutamatergic, NMDA, and GABAergic systems in the brain (Bear et al., 2004; Darnell et al., 2011; Davidovic et al., 2011; Paluszkiewicz et al., 2011), thereby impacting neuronal firing. This imbalance of the major excitatory and inhibitory neurotransmitter systems is thought to contribute to phenotypes in FXS cognitive and functional impairment and epilepsy (Paluszkiewicz et al., 2011). In the critical cortical period of development, a phase characterized by heightened neuronal plasticity beginning at birth and closing around postnatal day 14 (Lo et al., 2017), the switch of GABA neurons from depolarizing to hyperpolarizing is delayed in fragile X mice (He et al., 2014). During this period, the excitatory connection of the thalamocortical synapse is fundamentally disrupted in *Fmr1* KO mice in early postnatal development (Harlow et al., 2010). FMRP has been identified to directly target mRNA transcripts of multiple GABA_A_ subunits and GABA transporter *Slc6a1* (Miyashiro et al., 2003; Darnell et al., 2011; Ascano et al., 2012; Braat and Kooy, 2015; Braat et al., 2015), including lowered GABA_A_ receptor subunit mRNA and protein levels in both FXS mouse and *Drosophila* models (El Idrissi et al., 2005; D’Hulst et al., 2006; Gantois et al., 2006; Adusei et al., 2010). Levels of the GABA synthesizing enzyme, glutamic acid decarboxylase, have also been found to be affected in *Fmr1* knockout (KO) *Drosophila*. Lastly, recent studies reveal changes in GABAergic signaling at both pre- and post-synaptic domains of inhibitory synapses in an FXS mouse model (Kramvis et al., 2020). Hence, alterations in GABA_A_ receptor distribution and GABA production and metabolism are pertinent to FXS and make the GABAergic system an attractive path to study.

FXS researchers have used preclinical FXS models, like the *Fmr1* KO mouse (The Dutch-Belgian Fragile X Consortium, 1994; Dahlhaus, 2018) to develop novel therapeutic strategies that have found shown promise for clinical translation, however, many clinical trials employing these strategies, unfortunately, do not pass Phase 3 (Gross et al., 2015; Ligsay and Hagerman, 2016). A newly published preclinical study using gaboxadol (OV101), a GABA agonist, has shown reversal of FXS-related behavior such as hyperactivity, anxiety, and cognition (Cogram et al., 2019), and an OV101 phase 2 clinical trial has succeeded with positive results including improved anxiety and social withdrawal (NCT03697161). These studies indicate that a therapeutic approach addressing GABA in FXS patients may be a molecular approach to reducing many symptoms for patients with FXS. Additionally, there are both decreased GABA_A__1_ subunit expression in neonatal mouse brains, as early as postnatal day 5 (Adusei et al., 2010) and decreased GABA concentrations were also found in multiple brain regions of neonatal and young *Fmr1* KO mice (Davidovic et al., 2011; Braat et al., 2015) further substantiating the investigation of the GABAergic system for FXS treatment. Integrating multimodal imaging such as, MRI and positron emission tomography (PET), into preclinical FXS research could enable the visualization of chemical and protein changes within the GABAergic system of FXS subjects that may afford insight towards new treatment strategies. Moreover, utilizing non-invasive imaging as a tool to monitor and evaluate treatment efficacy can improve the accuracy of study outcome measures by observing real-time changes in the brain, rather than relying on treatment response questionnaires filled out by patients and caregivers.

^1^H-magnetic resonance spectroscopy (^1^H-MRS) is an MRI technique clinically used to study brain disease (Öz et al., 2014; Wilson et al., 2019), including the measurement of GABA in respect to autism spectrum disorder (Harada et al., 2011; Horder et al., 2018; Fung et al., 2020). ^1^H-MRS is capable of non-invasively measuring neurotransmitters and metabolites, *in vivo* and is typically utilized in conjunction with structural MRI (sMRI) to assess neurochemical composition within brain anatomical regions (Faghihi et al., 2017). Furthermore, ^1^H-MRS is the only non-invasive method capable of measuring *in vivo* GABA concentrations, with the most common method in humans being a spectral-editing approach known as MEGA-PRESS (Cochran et al., 2015). In animal models studied at higher magnetic fields, non-edited approaches, such as PRESS, are also viable (Horder et al., 2018). Employing this non-invasive analytical technique in the clinic to study brain disease and abnormal cognitive development brings new information *via* quantifying neurotransmitters and metabolites in tissues of interest.

To date, clinical studies using ^1^H-MRS to study GABA in ASD have found a decrease in GABA concentration in the frontal lobe of children with ASD (Harada et al., 2011) while, alternatively found an increase in the dorsal lateral prefrontal cortex in adults with ASD (Fung et al., 2020). PET imaging studies in participants with FXS have found a structurally-dependent decrease in GABA_A_ receptor binding potential primarily for the thalamus (D’Hulst et al., 2015) using ^11^C-Flumazenil (FMZ), a GABA_A_ antagonist that binds to the post-synaptic benzodiazepine site of the GABA_A_ receptor, between the α and γ subunits. This decrease in binding potential suggests a decreased receptor expression, consistent with D’Hulst et al.’s (2006) findings of decreased mRNA expression for several α and γ subunits in the cortex. Clinical ^1^H-MRS has demonstrated its feasibility to begin understanding the relationship between PET data indicating a decrease in GABA_A_ receptor density vs. the changes in free GABA neurotransmitter concentration in FXS. However, the previously mentioned confounding clinical ^1^H-MRS data for ASD warrants preclinical investigation of how the GABAergic system is affected by FXS concerning GABA concentrations. Increasing the use of non-invasive imaging in preclinical research can aid in determining the effectiveness of FXS therapy on a molecular level by being able to visualize changes in endogenous GABA levels *via* ^1^H-MRS. ^1^H-MRS can also be paired with PET to study how the GABA_A_ receptors may be altered by implementing novel FXS therapies. An advantage to utilizing preclinical non-invasive imaging for novel therapeutic validation is the ability to compare imaging results, particularly in longitudinal studies, with previously collected and validated *in vitro* and *ex vivo* data to gain a comprehensive understanding and correlation with functional molecular biology.

Since ^1^H-MRS can non-invasively measure GABA concentrations in both clinical and animal subjects, we sought to employ this technique to allow for future cross-species comparisons with *in vitro/ex vivo* experiments that are easily performed in relevant disease models. In particular, this study examined GABA levels in the thalamus and frontal cortex of 5-day-old *Fmr1* KO compared to WT mice. Aberrant neurodevelopment was exhibited in both regions of young children with FXS (Hoeft et al., 2010) and in the excitatory thalamocortical synapses in the somatosensory cortex of *Fmr1* KO mice during the critical cortical period (Harlow et al., 2010). Though it is reported that the perturbation in the GABAergic system is more substantial around day 12 toward the end of the critical cortical window (Adusei et al., 2010; Davidovic et al., 2011), our study concentrates on the earliest age (5-days-old) that GABAergic alteration has been detected (Adusei et al., 2010). Our strategy focuses on utilizing non-invasive imaging to capture the front end of the critical cortical period where abnormal development occurs due to loss of FMRP, to understand the changes in the brain that precedes the more substantial atypical development seen around day 12. The ^1^H-MRS GABA measurements were then compared to GABA levels in brain homogenates as quantified using liquid chromatography-tandem mass spectrometry (LC-MS/MS) to determine whether ^1^H-MRS is suitable for studying GABA levels in FXS. Validating ^1^H-MRS as a method to non-invasively assess GABA concentrations during early development would also provide a powerful tool to elucidate the role GABA plays in FXS and evaluate novel therapeutic strategies for clinical translation.

## Materials and Methods

### General

Unless stated otherwise, all supplies and equipment were purchased from commercial sources and used for these preclinical studies without modification.

### Animals

All animal experiments were approved by Stanford IACUC, with animals having access to food and water *ad libitum* while being kept under a 12-h light/dark cycle. Experiments were carried out using 5-day-old Wild type (FVB.129P2-Pde6b+ Tyr^c-ch^/AntJ, Jackson Labs) and *Fmr1* knockout (FVB.129P2-Pde6b+ Tyr^c-ch^ *Fmr1*^tm1Cgr^/J, Jackson Labs) male mice weighing 2–4 g.

### Magnetic Resonance Imaging

MRI at the Stanford Center for Innovation in *in vivo* Imaging (SC*i*3) includes an actively-shielded Bruker 7 Tesla horizontal bore scanner (Bruker Corp., Billerica, MA, USA), with International Electric Company (IECO) gradient drivers, a 120 mm inner diameter (ID) shielded gradient insert (600 mT/m, 1,000 T/m/s), AVANCE III electronics; eight-channel multi-coil RF and multinuclear capabilities; and the supporting Paravision 6.0.1 platform. Acquisitions were performed with an 86 mm ID actively de-couplable volume radiofrequency (RF) coil with a four-channel cryo-cooled receive-only RF coil.

### ^1^H-Magnetic Resonance Spectroscopy (^1^H-MRS)

**Figure 1:**
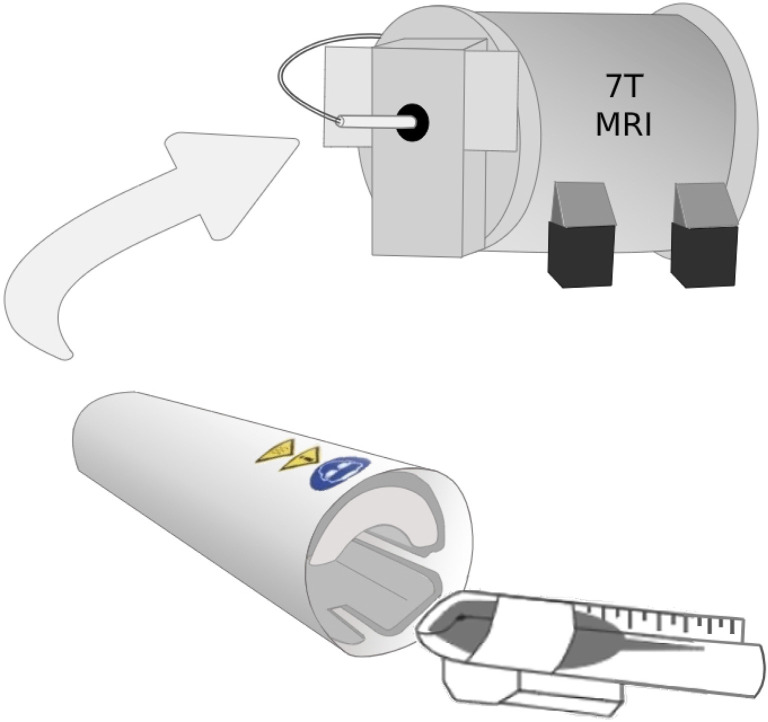
The 5-day-old mice were placed in a custom scanning bed that was created by cutting a 15 ml falcon tube lengthwise. After the mice were secured in the scanning bed with their nose in the conical portion of the tube to allow for airflow, the mice were inserted directly into CryoProbe and held in place by a hand-shaped piece of packing foam. The probe was inserted into the scanner with the mouse brain already in the isocenter of the probe.

Before each scan, WT (*N* = 10) and *Fmr1* KO (*N* = 5) mice were anesthetized using ketamine (45 mg/kg) and dexmedetomidine (0.9 mg/kg). ^1^H-MRS scans were then performed using a Bruker 7T animal MR scanner using a cryogenically cooled RF coil (CryoProbe, Bruker Instruments). Specifically, the 5-day-old mice were placed in a custom scanning bed that was inserted directly into CryoProbe before placing the probe into the scanner (Figure 1). A localizer is used to verify the mouse brain is in the isocenter of the probe. The mice were given subcutaneous saline before the scan and kept warm through heated airflow maintained at 37°C for the duration of the scan. Heart rate monitoring was not possible during the scan due to the small size of the mice used.

**Figure 2:**
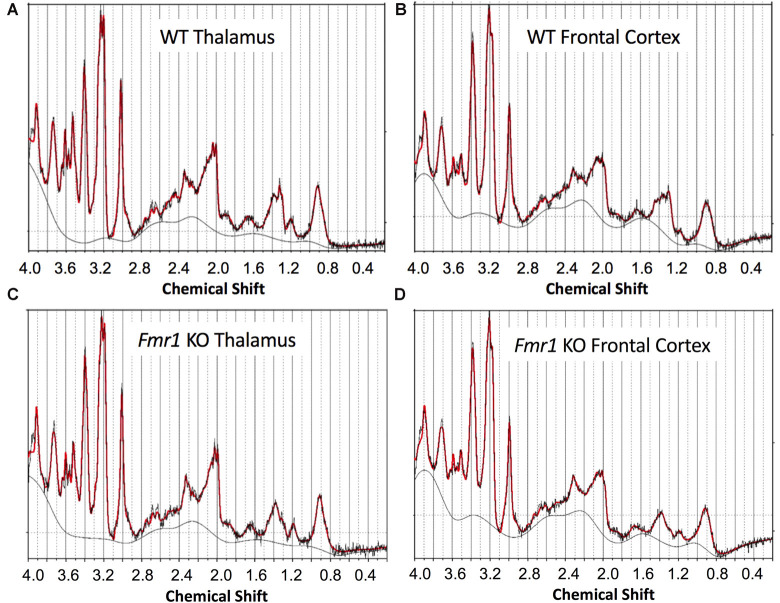
Representative *in vivo* ^1^H magnetic resonance imaging (^1^H-MRS) spectra of thalamus-rich and frontal cortex-rich voxels and corresponding fits of wild type and *Fmr1* KO 5-day-old mouse brain obtained at 7T with a Bruker CryoProbe and analyzed by LC Model. **(A)** WT thalamus region, **(B)** WT frontal cortex region, **(C)** *Fmr1* KO thalamus region, and **(D)** *Fmr1* KO frontal cortex region. Scan parameters: 2 × 2 × 2 mm^3^ voxels, TE = 16 ms, TR = 3,000 ms, spectral bandwidth = 5,000 Hz and 256 averages.

*In vivo* single-voxel spectroscopy using the PRESS sequence was then performed on thalamus-rich and frontal cortex-rich regions-of-interest in the mouse brain. Voxels were manually placed on thalamus-rich and frontal cortex-rich regions on a T2-weighted MRI anatomy scan with the following parameters: 2 × 2 × 2 mm^3^ voxels, TE = 16 ms, TR = 3,000 ms, spectral bandwidth = 5,000 Hz and 256 averages. The transmit gain was adjusted manually, and both field-mapped and localized voxel shimming were performed. Water suppression using the Vapor method in combination with outer volume suppression was also applied (Henning, 2018; Muñoz-Hernández and García-Martín, 2018). Finally, an unsuppressed-water spectrum was acquired from each voxel for magnetic field drifts and eddy current corrections. Figure 2 shows representative ^1^H-MRS spectra from wild type and *Fmr1* KO mouse brains. Spectral quantification and determination of metabolite ratios to total creatine (creatine + phosphocreatine, CR + PCR) were achieved using LCModel (Provencher, 2001). Basis sets were generated based on density matrix simulations of the sequence using values for chemical shifts and J-couplings of metabolites. LCModel fitting %SD values for our metabolite of interest GABA was ≤12%. LCModel is a commercial software that quantifies metabolites from an MRS spectrum using a least-squares combination of basis spectra. The level of confidence was reported as %SD with 20% or less considered acceptable in general. The basis set is simulated for TE = 16 ms at 7T and contains metabolites of interest.

### Liquid Chromatography-Tandem Mass Spectrometry (LC-MS/MS)

Brains from a separate group of mice were harvested from WT (*N* = 5) and *Fmr1* KO (*N* = 5) mice following decapitation. The frontal cortex and thalamus were collected in microfuge tubes using the Palkovits Punch technique and immediately frozen on dry ice (Palkovits, 1973). The tissue was then homogenized in PBS at a concentration of 150 μl PBS per gram of tissue, and the analyte was extracted from the tissue homogenate using cold 0.1% formic acid in acetonitrile. The sample extracts were derivatized according to Ez:faast™ Amino Acid Analysis protocol according to the manufacturer’s instructions (Phenomenex, Torrance, CA, USA), dried under nitrogen, and reconstituted in LC-MS/MS buffer before the analysis. The LC-MS/MS system consisted of an HP1100 HPLC stack (Agilent, Santa Clara, CA, USA) coupled to a Quattro Premier triple quadrupole mass spectrometer (Waters, Milford, MA, USA). The LC system includes a binary pump, degasser, temperature-controlled column compartment, and autosampler. The mass spectrometer was equipped with an electrospray ionization (ESI) source. The LC separation was achieved on EZ:faast™ AAA-MS column (250 × 3 mm, KH0-7338, Phenomenex, Torrance, CA, USA) at 35°C. Mobile phase A (10 mM ammonium formate) and mobile phase B (10 mM ammonium formate in methanol) were used for a gradient elution from 68 to 83% B at a flow rate of 250 μl/min with a total LC-MS/MS run of 17 min. The mass spectrometer was operated in positive mode using selected reaction monitoring (SRM) scanning mode. The protonated molecules of GABA (m/z 232.0, A2129, Sigma–Aldrich, St. Louis, MO, USA) and GABA-*d6* (m/z 237.9, D-1828, C/D/N Isotopes Inc., Quebec, QC, Canada) were used as precursor ions for collision-induced dissociation (CID) for MS/MS analysis. A unique precursor ion-fragment ion SRM transition was select for GABA (232.0 > 130.0, 172.1) and GABA-*d6* (237.9 > 135.7, 178.1) which was used as internal standard (IS). Obtained data were processed using QuanLynx software (Waters, Milford, MA, USA). Data were then normalized to protein concentration for each sample using a protein concentration assay (Pierce™ BCA Assay, Thermo Fisher Scientific, Waltham, MA, USA, Supplementary Table 1). Calibration curve standards covered the range of concentrations from 10 nM to 10 μM and resulted in a linear response over the tested range. The low limit of quantitation for GABA was 50 fmols.

### Statistical Analysis

GABA was measured in the thalamus and cortex of wild type (*N* = 10 ^1^H-MRS, *N* = 5 LC-MS/MS) and *Fmr1* knockout (*N* = 5 ^1^H-MRS, 5 LC-MS/MS) mice, using either ^1^H-MRS or LC-MS/MS methods. For each method a separate linear regression of GABA on factors of type (*Fmr1* KO vs. WT) and region (cortex vs. thalamus) was done, using a robust Huber-White (Huber, 1967; White, 1980) variance estimator to adjust for clustering within the same animal.

## Results

Endogenous GABA levels were measured *in vivo* and normalized to total creatine (CR + PCR) using ^1^H-MRS. Thalamic and frontal cortical GABA concentrations were compared cross-structurally (within each mouse) and between the same structure across WT and *Fmr1* KO mouse groups. To validate the preclinical model used, we employed LC-MS/MS to provide another method for measuring the GABA concentrations *in vitro* and demonstrate a change in GABA concentration between animal cohorts. Specific dissections for the thalamus and frontal cortex from both WT and *Fmr1* KO mice were made and processed for LC-MS/MS analysis. Endogenous GABA concentration measurements were obtained and subsequently compared between both regions within the same animal and for each region across both groups.

**Figure 3:**
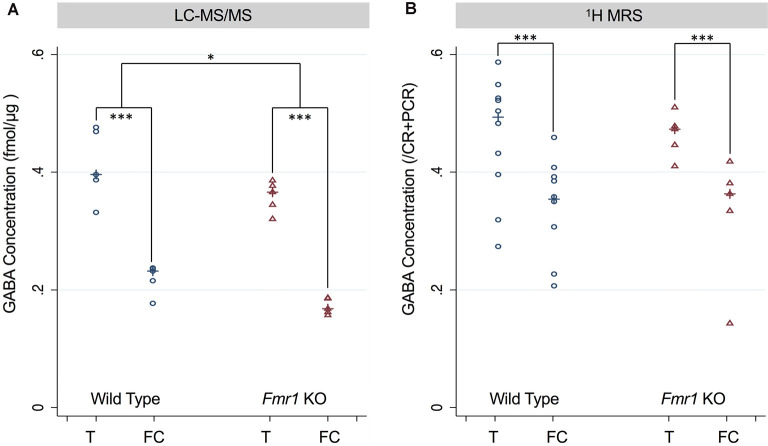
**(A)** GABA concentration measured in the thalamus and frontal cortex of P5 wild type (WT) and *Fmr1* KO mice measured using LC-MS/MS and normalized to the protein concentration of the sample. **(B)** GABA concentration measured using ^1^H-MRS GABA/CR + PCR ratios from thalamus-rich and frontal cortex-rich regions of P5 wild type and *Fmr1* KO mice analyzed by LC Model. Statistical significance was determined using a robust Huber-White variance estimator. *p < 0.05, ***P < 0.001.

For both LC-MS/MS and ^1^H-MRS methods, both WT and *Fmr1* KO mice demonstrated within in each animal statistically significantly higher GABA levels (*p* < 0.001, Figure 3) in the thalamus-rich region compared to frontal cortical-rich regions. With LC-MS/MS methods, WT mice had statistically significantly higher GABA concentration than *Fmr1* KO mice (*p* = 0.028, Figure 3A), but we were unable to detect a statistically significant difference in GABA concentration between WT and *Fmr1* KO groups by ^1^H-MRS methods (*p* = 0.92, Figure 3B).

## Discussion

Current treatment methods for children and adults with FXS focus on alleviating the symptoms of FXS, such as ADHD and decreased social function, vs. treating the root cause (i.e., loss of FMRP and how the brain is affected). In a clinical trial, social functioning was improved by the use of intranasal oxytocin (Hall et al., 2012) and symptoms of ADHD were improved with the use of pharmaceutical stimulants, such as methylphenidate (Hagerman et al., 1988). While improvements in symptomatic molecular treatments are valuable, a new molecular target may provide an opportunity to improve the quality of life for patients and their families and novel approaches to preclinical FXS research are imperative. Exploring non-invasive imaging strategies [i.e., PET, sMRI, functional MRI (fMRI), diffusion tensor imaging (DTI), MRS] to study biological systems (i.e., GABAergic) may empower researchers to see how novel therapies change the brain early in development and understand the underlying mechanisms leading to disease phenotypes (i.e., FXS).

This study, to the best of our knowledge, is the first ^1^H-MRS and LC-MS/MS GABA study performed on 5-day-old mouse brains from the *Fmr1* KO mouse model to investigate the impact FMRP loss has on endogenous GABA concentration in the frontal cortex and thalamus. Our ^1^H-MRS study detected significantly decreased GABA in the frontal cortex relative to that observed in the thalamus of both the wild type and *Fmr1* KO mice and confirmed these measurements using LC-MS/MS. The development of our method to measure these extremely small animals in a cryogenically cooled RF coil (Bruker CryoProbe) on a 7T MRI required the reliable placement of the neonatal brain in the isocenter of the scanner. Moreover, positioning the neonates on the heated ceramic casing within the CryoProbe ensures consistent warmed body temperature during the MRI scan. Additionally, the use of the CryoProbe increases SNR by reducing the noise from the coil itself, caused by resistance in the coil’s wiring (Ratering et al., 2008; Baltes et al., 2009). Our novel scanning technique can be applied to any mouse model for MRI scanning, including sMRI, ^1^H-MRS, fMRI, and DTI. Furthermore, our technique uses a homemade scanning bed constructed from common laboratory materials (Figure 1), making this method of scanning neonates using a CryoProbe easily reproducible.

Although ^1^H-MRS was unable to detect the same-structure differences within the frontal cortices or thalami between *Fmr1* KO mice and WT mice, LC-MS/MS was able to detect significantly lower GABA in the frontal cortex and thalamus of *Fmr1* KO mice compared to age-matched WT mice. This is the first report of lowered GABA concentration in the frontal cortex and thalamus in a 5-day-old FXS mouse model. Alterations within these structures are supported by previous clinical imaging studies showing abnormal activation patterns in the supplementary motor area of the cerebral cortex and thalamus in female FXS patients *via* fMRI (Menon et al., 2004) and significantly decreased binding potential in the thalamus of male FXS patients found with ^11^C-FMZ PET (D’Hulst et al., 2015). Overall, the findings of these imaging studies in combination with our detection of decreased GABA in the thalamus supports that FMRP loss affects multiple components of the thalamus, including the GABAergic system. Recent studies have also found that inhibitory deficits in the medial prefrontal cortex in a prepubescent FXS mouse model (Kramvis et al., 2020). Multiple clinical ^1^H-MRS studies measuring GABA in various adult populations have found that decreased GABA concentrations in the prefrontal cortex, dorsolateral prefrontal cortex, or frontal cerebrum are correlated with decreased cognitive function and working memory task performance (Yoon et al., 2016; Porges et al., 2017; Kim et al., 2019). Another ^1^H-MRS clinical study found a positive correlation between visual intelligence and GABA concentration in the primary visual cortex (Cook et al., 2016). These results are consistent with our studies showing lowered GABA concentration in the frontal cortex, as decreased cognition and lowered IQ are characteristic of FXS.

This study confirmed that *in vivo* ^1^H-MRS is a promising non-invasive imaging technique for detecting GABA-regional brain differences within 5-day-old mice of each respective cohort, despite not detecting structural differences between *Fmr1* KO vs. WT mice in this study. To use similar ^1^H-MRS methods to detect smaller differences in GABA concentration between *Fmr1* KO and WT mice detected by LC-MS/MS improved MRI sensitivity (using techniques such as increased averaging, larger voxels, or higher magnetic gradients) to increase the signal to noise ratio (SNR) or more animals would be needed for the study (i.e., increased sample size). Based on the linear regression models’ marginal effect of type, the standard error for LC-MS/MS mice is 0.019, while that for ^1^H-MRS mice is twice as large, 0.039, even though the sample size is 50% larger in number. To achieve a similar standard error to that of the LC-MS/MS mice, the 1H MRS study would require a sample size of 65 animals. The apparent variability in the ^1^H-MRS GABA measurements, particularly for each animal in the WT group (Figure 3B), may contribute to the lack of significant difference seen in same-structural comparisons between WT and *Fmr1* KO mice. Since decreased GABA transmission in either the medial prefrontal cortex or basolateral amygdala has been linked with decreased sociability, this observed variability may be due to a wider range in social function in control animals when compared to animals with known social deficits. Changes in GABA signaling is seen in conditions, such as autism spectrum disorder, may mediate the social withdrawal aspect of these conditions (Paine et al., 2017). Additionally, our voxel size and cubic shape of 2 × 2 × 2 are larger than the volume for the frontal cortex and thalamus of our 5-day-old mice, thus increasing the variability in the ^1^H-MRS data. In particular to our study of GABA, we chose to use separate animals for each technique because the animals that underwent ^1^H-MRS scanning were subjected to anesthesia. Anesthesia has a known effect on both circadian rhythms (Poulsen et al., 2018) and the GABA_A_ receptor’s binding to ^18^F-FMZ (Palner et al., 2016) therefore, we chose to eliminate anesthesia from our *in vitro* LC-MS/MS analysis. Another factor to consider is that GABA exists in different states *in vivo* including bound, free, and within vesicles. These different pools of GABA could have varying relaxation times and, hence, may have differential sensitivity to detection using ^1^H-MRS. Conversely, for LC-MS/MS studies, the brain sections are homogenized, likely releasing all available GABA.

If higher SNR can be achieved, ^1^H-MRS could be a powerful non-invasive imaging tool for measuring endogenous FXS GABA levels, which could potentially be paired with ^11^C- or ^18^F-FMZ PET to image the postsynaptic benzodiazepine site on the GABA_A_ receptor. Such experiments would allow the assessment of correlations between regional GABA concentrations and receptor densities between WT and *Fmr1* KO mice. Having a better understanding of how neuronal GABA varies between WT and *Fmr1* KO mice could provide new information into the mechanisms of FXS’s impact on early-stage brain development. Implementing ^1^H-MRS into pre-clinical therapeutic evaluation studies in an FXS model can be particularly valuable for longitudinal studies.

Our study is the first known ^1^H-MRS and LC-MS/MS measurements of GABA in 5-day-old mice. We demonstrated that a Bruker 7T MRI with a cryogenically cooled RF coil provides sufficient sensitivity to measure regional significant differences in GABA concentration between the frontal cortex and thalamus within individual 5-day-old mice. However, the sensitivity to see significant differences within the same structures between WT and *Fmr1* KO mice did not measure up to the LC-MS/MS being able to detect a significant decrease in both structures in the *Fmr1* KO mice compared to WT. This *in vitro* data elucidates that alterations in the GABAergic system of 5-day-old FXS mice extend further than the previously reported subunit alterations (Adusei et al., 2010). Although LC-MS/MS is a valuable *in vitro* technique that can be used to complement *in vivo* data, it is not ideal to analyze brain samples from animals studied over many time points. Exploring novel FXS therapy approaches tailored to the critical window of cortical development may have promising outcomes due to addressing changes in the GABAergic system at their earliest occurrence. For ^1^H-MRS to be routinely utilized in preclinical research and development of novel FXS molecular therapeutics, our studies suggest an increased field strength or increased scan time can be used to improve SNR while employing a cryogenically cooled RF coil.^ 1^H-MRS is non-invasive and does not require contrast agents or radiotracers, making it an attractive modality to use for longitudinal studies. ^1^H-MRS can be an incredibly useful tool to monitor the effectiveness of novel therapies at addressing how to normalize the concentration of free neurotransmitters, such as GABA in the brain.

## Data Availability Statement

The original contributions presented in the study are included in the article/Supplementary Materials, further inquiries can be directed to the corresponding author.

## Ethics Statement

The animal study was reviewed and approved by Stanford Institutional Care and Use Committee (IACUC).

## Author Contributions

FC and DS conceived the experiments. FC, DS, SR, SM and LP designed the 1H-MRS experiment. FC, DS, SR and KK designed the LC-MS/MS experiment. All authors performed the experiments and/or analyzed the data. SR wrote the manuscript with the support of all authors. All authors contributed to the article and approved the submitted version.

## Conflict of Interest

The authors declare that the research was conducted in the absence of any commercial or financial relationships that could be construed as a potential conflict of interest.
