# Supplementary material for: GABA Measurement in a Neonatal Fragile X Syndrome Mouse Model Using 1H-Magnetic Resonance Spectroscopy and Mass Spectrometry
Source: Front Mol Neurosci. 2020 Dec 18;13:612685. doi: 10.3389/fnmol.2020.612685 (PMC7775297; doi:10.3389/fnmol.2020.612685)
Supplement: Supplementary file 1 [file Table_1.pdf]

**Table S1**. This table shows the protein concentrations for all the samples measured by LC-MS/MS, the averaged raw LC-MS/MS values and the normalized GABA concentrations (LC-MS/MS measured GABA concentration divided by protein concentration of each sample).

| Animal Genotype | Animal Number | Structure | Protein Concentration from Assay (µg/mL) | Protein Concentration (µg/uL) | Average LC-MS/MS GABA Concentration (fmol/µL) | Normalized GABA Concentration (fmol/µg) |
|---|---|---|---|---|---|---|
| *Fmr1* Knockout | 1 | Thalamus | 221.8333 | 0.2218 | 8357.6000 | 37675.1315 |
| *Fmr1* Knockout | 1 | Frontal Cortex | 201.5000 | 0.2015 | 3757.9333 | 18649.7932 |
| Wild type | 1 | Thalamus | 211.6111 | 0.2116 | 8378.7667 | 39595.1168 |
| Wild type | 1 | Frontal Cortex | 324.0556 | 0.3241 | 6992.5333 | 21578.1930 |
| *Fmr1* Knockout | 2 | Thalamus | 279.5556 | 0.2796 | 10788.3000 | 38590.8983 |
| *Fmr1* Knockout | 2 | Frontal Cortex | 335.2222 | 0.3352 | 5440.3000 | 16228.9360 |
| Wild type | 2 | Thalamus | 269.6111 | 0.2696 | 12842.0333 | 47631.6917 |
| Wild type | 2 | Frontal Cortex | 308.2778 | 0.3083 | 7154.7333 | 23208.7223 |
| *Fmr1* Knockout | 3 | Thalamus | 178.5556 | 0.1786 | 6534.5333 | 36596.6397 |
| *Fmr1* Knockout | 3 | Frontal Cortex | 292.1111 | 0.2921 | 4590.6667 | 15715.4812 |
| Wild type | 3 | Thalamus | 207.1111 | 0.2071 | 6867.8667 | 33160.3004 |
| Wild type | 3 | Frontal Cortex | 317.0556 | 0.3171 | 5616.0000 | 17712.9841 |
| *Fmr1* Knockout | 4 | Thalamus | 151.3333 | 0.1513 | 5212.4667 | 34443.6123 |
| *Fmr1* Knockout | 4 | Frontal Cortex | 369.1667 | 0.3692 | 6848.2667 | 18550.6095 |
| Wild type | 4 | Thalamus | 211.0000 | 0.2110 | 8165.7667 | 38700.3160 |
| Wild type | 4 | Frontal Cortex | 323.1667 | 0.3232 | 7670.5000 | 23735.4306 |
| *Fmr1* Knockout | 5 | Thalamus | 245.5000 | 0.2455 | 7861.4000 | 32021.9959 |
| *Fmr1* Knockout | 5 | Frontal Cortex | 332.4444 | 0.3324 | 5587.0333 | 16805.9158 |
| Wild type | 5 | Thalamus | 120.1111 | 0.1201 | 5632.4000 | 46893.2470 |
| Wild type | 5 | Frontal Cortex | 292.7778 | 0.2928 | 6910.3000 | 23602.5427 |
